# Assessment of parental nurturing and associated social, economic, and political factors among children in the West Bank of the occupied Palestinian territory (WB/oPt)

**DOI:** 10.1186/s12887-020-02317-0

**Published:** 2020-08-29

**Authors:** Nouh Harsha, Luay Ziq, Margaret A. Lynch, Rita Giacaman

**Affiliations:** 1 Department of Public Health and Epidemiology, Faculty of Medicine, University of Debrecen, Debrecen, Hungary; 2 Institute of Community and Public Health, Birzeit University, ICPH/BZU, Birzeit, Palestine; 3 MPH, ICPH/BZU, UNRWA, Ramallah, West Bank Palestine; 4 Kings College, London, UK; 5 ICPH/BZ, Birzeit, Palestine

**Keywords:** Parental nurturing, Emotional warmth, Palestinians on the West Bank, Discipline, Child disability, Political violence

## Abstract

**Background:**

Parental nurturing expressed through love and affection is a broad concept that entails caring for children and their activities, encouraging them and praising their achievements. Lack of love and affection makes children more susceptible to psychological problems such as stress, anxiety and depression across their life time. This study aims to evaluate parental nurturing and associated social, economic, and political factors among Palestinian children living in the West Bank (WB).

**Methods:**

Secondary data representative of the Palestinian children living in the WB was used to estimate parental nurturing for children aged 0–12 years as reported by their mothers. Univariate and bivariate analyses were conducted, followed by multivariate analysis for all predictors found significant in the bivariate analysis using SPSS® version 20.

**Results:**

19.90% (231/1162) of children experienced low levels of parental nurturing. No statistically significant differences were detected by the child’s gender. Children with high levels of parental nurturing were those aged 0–6 years, children who were last in the family index, children with no disability, children exposed to low to medium levels of disciplinary methods, children from urban areas, children living in North WB, and children whose families were not subjected to political violence.

**Conclusions:**

Overall, Palestinian mothers reported high levels of parental nurturing towards their children. However, about one-fifth of Palestinian children are at risk of experiencing low levels of parental nurturing. Efforts should be placed in addressing the health and welfare needs of these high-risk children’s groups.

## Background

Parenting refers to the activities of raising the children to enable them to survive, grow, and develop to their maximum potential [1]. The literature identifies two dimensions for parenting: discipline and nurturance [2]. Parental nurturing or warmth that includes both maternal and paternal warmth is a broad notion entailing caring for children, their friends and their activities, encouraging them, supporting them, involving them and praising their achievements [3, 4]. Parental nurturing forms the base for the emotional and social means through which children develop trust, self-confidence, security feeling and competence [5, 6].

Parental nurturing is a cornerstone in the formation and development of personality and psychology of children and has received considerable research attention [7]. Several studies demonstrate a negative association between low parental nurturing and social behavior, academic success, and work achievement [8, 9]. Researchers found a positive association between school achievement and positive parenting practices which reflect the specific behaviors used by parents to raise their children such as reward and punishment [10–12]. Parenting practices play an important role in protection against internalizing behaviors such as anxiety and depression as well as externalizing behaviors such as aggression [13]. Russell has examined the relationship between child misbehavior and the positive parental interaction and reported that love and affection are negatively associated with children’s misbehavior and hostility [14]. Lack of love and affection and poor parent child relationship has been shown to make children more susceptible to psychological disorders such as stress, anxiety and depression [4, 15].

Several factors have been reported to affect parental nurturing and affection. Those include child characteristics such as age, gender, physical and mental health and disability [16, 17]. In addition, family socioeconomic status, the child’s ordinal position in the family [10, 18–20], family history and life situation, cultural beliefs and way of child rearing or parenting [21, 22], marital status and parental conflicts are major determinants [23, 24]. In a study conducted to determine predictors of parenting during early childhood Waylen and Brown reported that good maternal health and social support were positively related to high parental nurturing [25]. Furthermore, other research reported a negative association between use of corporal punishment and levels of emotional warmth [26]. Poor parent child relationship and domestic violence were found to increase risks of child maltreatment [27]. In 2013, Carroll and colleagues reported a significant inverse association between child abuse and parental warmth [28]. Overall, social, economic, and political factors are major determinants of quality of parental nurturing [7].

Children are sensitive to the surrounding environment and more likely to develop psychological distress due to domestic violence [29] and political turmoil [30]. Disruption of social, economic, and political aspects of life have been reported in areas experiencing conflict, fragility, and instability [31]. This toxic environment and the resulting social and individual stress are likely to reduce capacity of the parents to provide optimal nurturing and care for their children which is necessary for their wellbeing and development [7].

In the occupied Palestinian territory (oPt), there is a chronic lack of political stability [32]. A study conducted in 2015 by the Palestinian Central Bureau of Statistics (PCBS) in collaboration with UNICEF reported that 92.2% of the Palestinian children aged 1 to 14 years experienced one or more forms of physical or psychological violence in the month preceding the survey [33]. Given that parental nurturing plays an important role in child psychological wellbeing [34] and in neutralizing effects of political and domestic violence [30, 35], and because Palestinians continue to experience chronic and protracted political violence with negative economic and social effects, studying and understanding parental nurturing in the oPt is essential.

A study conducted in 9 countries -including Jordan, a country with similar social, religious, and cultural profile to the Palestinian context, and Colombia which has undergone decades of political instability- to assess parental warmth, acceptance and hostility towards children concluded that parents demonstrated high levels of warmth and acceptance in all countries studied [36]. To our knowledge, this is the first study on parental nurturing in the oPt at the national level. This study aims to evaluate levels of parental nurturing among Palestinian children aged 0–12 years living in the West Bank (WB) and to determine its selected social, economic, and political associated factors. We hypothesize that (1) the Palestinian mothers of the WB exhibit high levels of parental nurturing, (2) child discipline is inversely associated with parental nurturing, and (3) parental nurturing has social, economic and political associated factors.

## Methods

### Data source

We used secondary data representative of Palestinian children living in the WB of the oPt based on the Palestinian Census Projections of 2007. Data were extracted from a large cross-sectional survey conducted to compare children’s disciplinary methods used by mothers/caregivers in Palestine and Qatar at the national level in 2014/2015 using the International Society for Prevention of Child Abuse and Neglect (ISPCAN) Child Abuse Screening Tool for parents (ICAST-P) tool [37]. 1299 mothers were interviewed with a response rate of 94.4%. Data collection was completed during November and December of 2014.

### Instrument

The International Society for Prevention of Child Abuse and Neglect (ISPCAN), with the support of UNICEF and other international bodies, developed the Child Abuse Screening Tools (ICASTs) to assess the global prevalence of violence against children. Three independent questionnaires were developed: ICAST-P (parents version); ICAST-C (children version); and ICAST-R (retrospective reporting) [38]. The ICAST-P questionnaire which covers the most frequently used disciplinary practices worldwide was used to assess disciplinary practices against Palestinian children aged 0 to 12 years old as reported by their mothers. The ICAST-P questionnaire is a useful tool for identifying and comparing the disciplinary practices that are used in different contexts and cultures and can be used for children up to 18 years [39]. Studying parental nurturing at early age is essential for promoting early child development and achieving maximum potential [40].

### Inclusion and exclusion criteria

The study was inclusive to children aged 0 to 12 years. Mothers aged 16 years and above were recruited and interviewed. This study is representative of the Palestinian children living the WB only. The Qatari data were not included in this study.

### Outcome measured

Mothers were asked three questions to measure parental nurturing. The questions were about child being loved by his parents during the last 12 months (Appendix 1). Question consistency was checked and Cronbach’s alpha for the scale was very good (0.84). In addition, factor analysis was conducted and showed that all the questions are loaded on a single component. Answers were rated using a Likert scale consisting of six points (1 = more than 10 times, 2 = six to 10 times, 3 = three to five times, 4 = once or twice, 5 = not in the last year, 6 = never). The scale was recoded as 0 (no, never) and 4 (more than 10 times). The nurturing scale was built by computing the three variables to obtain a scale from 0 (minimum nurturing) to 12 (maximum nurturing). We multiplied the scale by 100/12 to get a 100 point scale.

Several statistical approaches use different cut-off points to create categorical variables [41–43]. Given that the calculated outcome here is high (Mean = 91.9; SD ±15.4), we used the statistical formula mean ± 0.25 standard deviation [44] to calculate the cut-off points since no standard cut-off point has been identified for the West Bank, and because this method is used in such situations. Based on this, we obtained three categories: low levels for those who scored below 88%; medium levels for those who scored between 88 and below 96%; and high levels for those who scored 96% and above. Given that we are interested in investigating the category of most disadvantaged children (children with low levels of nurturing) we combined the other two categories (medium levels and high levels) together to form a single category. Thus, we obtained the two categories dependent variable: low levels versus medium to high levels of nurturing using the cutoff point 88%.

### Independent variables

The selected independent variables included were the ones recommended by the IPSCAN tool in addition to other important variables specific to the oPt. The original study was a joint one of two countries (Qatar and oPt) with a limit to the number of variables which could be included to ensure that the questionnaire would not take too long to fill and be acceptable to mothers.

The independent variables investigated were as follows: child’s gender; child’s age categorized into 3 categories (below 6 years, 6–9 years, and 10 to 12 years); family size categorized into 3 categories (families with 4 members or less, families with 5 to 7 members, and families with 8 or more members); child’s index to indicate the ordinal position of the child within the family as the first or only child, middle child, or the last child in the family; the crowding ratio at home obtained by dividing the number of residents by the number of the rooms (two person or less and more than two persons per room); educational levels of parents (less than secondary, secondary, and post-secondary); their employment status (mothers were classified as employed or unemployed/housewife, and fathers were categorized as laborer, middle class manager/landowner, or unemployed); number of persons working in the family (none or 1–4 persons); child’s disability categorized into two categories (with no disability or with one or more disabilities); use of disciplinary methods (low to medium versus high levels); locality type were the family resides (urban, rural, or camp); region where the family lives in the West Bank (north, center, or south); refugee status (refugee or non-refugee); and parents’ exposure to political violence in the form of tear gas or/and sound bombs (whether the parents were exposed or not).

For disciplinary methods, a scale was made out of 28 items representing both physical as well as psychological methods most commonly used across the globe (Appendix 2). Cronbach’s alpha for discipline scale was 0.82 indicating very good internal consistency. We used a six point Likert scale (categorized as 1 = once or twice, 2 = 3–5 times, 3 = 6–10 times, 4 = more than 10 times, 5 = not in the past year, 6 = never) to report mothers’ responses for each question. Responses obtained were recoded as 0 (never or not in the past year) and 1(one to 10 times). The scores were aggregated to form a scale from 0 (minimum discipline) to 25 (maximum discipline). The scale was recoded using the formula mean ± 0.25 standard deviation [44] into three categories: low levels for those who experienced 6 disciplinary methods or less; medium levels for those who experienced more than 6 and less than 9 disciplinary methods; and high levels for those who experienced 9 disciplinary methods and above. Since we are interested in investigating the most disadvantaged category (children exposed to high levels of disciplinary methods) we combined the other two categories (low levels and medium levels of discipline) to a single category. Thus, those who experienced eight disciplinary methods or less were considered to be with low to medium levels discipline and those experienced nine methods or more were considered to be with high levels of discipline.

### Statistical analyses

The dependent variable was parental nurturing recoded into two categories: low levels versus medium to high levels of nurturing. Univariate analysis was completed with all the independent variables included in the study to obtain the basic characteristics of the sample. Bivariate analysis was completed to assess associations between parental nurturing and various independent variables using the Chi-square test. Variables found significant in cross tabulation were inserted into the binary logistic regression model. We set the level of significance at 95% (*P* < 0.05) using SPSS® version 20 in data analyses.

## Results

Table 1 below shows the basic characteristics of the study sample. 52.5% were males, 53.0% were less than 6 years old, 24.6% of the families had 8 members or more, 61.3% of children were last in the family index, 22.5% of the mothers reported post-secondary education, 10.5% of the children were with one or more disabilities, 33.0% experienced high levels of discipline, 37.8% were living in urban areas, 45.5% were living in North WB and 41.0% were children whose families were exposed to political violence.

**Table 1:** Descriptive characteristics of the study sample

Variable		Number	Percentage (%)
Gender (child)	Male	610	52.5
	Female	552	47.5
Age (years)	Less than 6	616	53.0
	6 to 9	404	34.8
	10 to 12	142	12.2
Family size	Up to 4	193	16.6
	5 to 7	683	58.8
	8 and more	286	24.6
Family child born index	Only child/first child	117	10.1
	Middle child	333	28.7
	Last child	712	61.3
Education (mother)	Less than secondary	674	58.0
	Secondary	227	19.5
	Post-secondary	261	22.5
Education (father)	Less than secondary	703	60.5
	Secondary	208	17.9
	Post-secondary	251	21.6
Employment (mother)	Employed	103	8.9
	Housewife and unemployed	1059	91.1
Employment (father)	Laborer	356	30.7
	Middle class manager/landowner	683	58.9
	Unemployed	121	10.4
Number of persons working part-time at household	No body	981	84.4
	1 to 4 persons	181	15.6
Crowding ratio	Two persons or less	771	66.4
	More than two persons	391	33.6
Child disability	No	1040	89.5
	Yes	122	10.5
Child discipline	Low to medium	779	67.0
	High	383	33.0
Locality type	Urban	439	37.8
	Rural	637	54.8
	camp	86	7.4
Region	North WB	529	45.5
	Center WB	266	22.9
	South WB	367	31.6
Refugee status	Refugee	350	30.1
	Non refugee	812	69.9
Political violence	No	686	59.0
	Yes	476	41.0

19.9% of the children experienced low levels of parental nurturing (Table 2). Mean level of parental nurturing was 91.9 (SD ±15.4) on a 100-point scale. No significant differences were detected by child gender. Parental nurturing varied by children’s age group: 16.2% of children aged less than six years; 20.5% of children aged 10 to 12 years, and 33.8% of children aged 10 to 12 years experienced low levels of parental nurturing (*p* < .05). 18.9% of nondisabled children had low levels of parental nurturing compared to 27.9% of children with one or more disabilities (*p* < .05). 14.8% of children subjected to low to medium levels discipline had low levels of parental nurturing compared to 30.3% of children subjected to high levels discipline (*p* < .05). 14.7% of children whose families were not subjected to political violence had low levels of parental nurturing compared to 27.3% of children whose families experienced political violence (*p* < .05). When we crosstabulated the region (north, center, and south) with education, employment, and family size, higher educational levels of mothers and higher employment rates in the family, and lower family sizes were noted for the North compared to the South WB).

**Table 2:** Parental nurturing levels by the predictor variables

Variable (n)		Parental nurturing (%) Low levels	Parental nurturing (%) Medium to high levels	Chi square (*P*-value)
Parental nurturing (1162)		19.9	80.1	
Gender (child)	Male (610)	19.8	80.2	0.002 (0.513)
	Female (552)	19.9	80.1	
Age (years)	Less than 6 (616)	16.2	83.8	
	6 to 9 (404)	20.5	79.5	22.54 (<0.001)
	10 to 12 (142)	33.8	66.2	
Family size	Up to 4 (193)	14.5	85.5	
	5 to 7 (683)	19.6	80.4	6.76 (0.034)
	8 and more (286)	24.1	75.9	
Family child born index	Only child/first child (117)	23.9	76.1	
	Middle child (333)	33.6	66.4	63.28 (<0.001)
	Last child (712)	12.8	87.2	
Education (mother)	Less than secondary (674)	22.4	77.6	
	Secondary (227)	22.0	78.0	14.88 (0.001)
	Post-secondary (261)	11.5	88.5	
Education (father)	Less than secondary (703)	23.3	76.7	
	Secondary (208)	17.8	82.2	15.73 (<0.001)
	Post-secondary (251)	12.0	88.0	
Employment (mother)	Employed (103)	6.8	93.2	12.15 (<0.001)
	Housewife/unemployed(1059)	21.2	78.8	
Number of persons working part-time at household	No body (981)	20.8	79.2	3.32 (0.040)
	1 to 4 persons (181)	14.9	85.1	
Crowding ratio	Two persons or less (771)	16.3	83.7	18.00 (<0.001)
	More than two persons (391)	26.9	73.1	
Child disability	No (1040)	18.9	81.1	5.46 (0.016)
	Yes (122)	27.9	72.1	
Child discipline	Low to medium (779)	14.8	85.2	38.85 (<0.001)
	High (383)	30.3	69.7	
Locality type	Urban (439)	14.4	85.6	
	Rural (637)	22.8	77.2	14.29 (0.001)
	Camp (86)	26.7	73.3	
Region	North WB (529)	9.8	90.2	
	Center WB (266)	27.4	72.6	61.78 (<0.001)
	South WB (367)	28.9	71.1	
Political violence	No (686)	14.7	85.3	27.96 (<0.001)
	Yes (476)	27.3	72.7	

Multivariate binary logistic regression using the categorized parental nurturing as the dependent variable is presented below (Table 3). No differences were detected by gender. Children aged 0–6 years were more likely to have high levels of parental nurturing compared to older children [OR = 3.07, 95%CI (1.90–4.96)]. Children who were last in the family were more likely to have high levels of parental nurturing compared to first or only child in the family index [OR = 2.20, 95%CI (1.20–4.03)]. Children with no disability were more likely to have high levels of parental nurturing compared to children with one or more disabilities [OR = 1.95, 95%CI (1.17–3.25)]. Children exposed to low to medium levels of discipline were more likely to have high levels of parental nurturing compared to children subjected to high levels of discipline [OR = 2.16, 95%CI (1.54–3.03)]. Children from urban areas were more likely to have high levels of parental nurturing compared to children from Palestinian refugee camps [OR = 2.25, 95%CI (1.18–4.29)]. Children living in North WB were more likely to have high levels of parental nurturing compared to children living in South WB [OR = 4.59, 95%CI (3.04–6.92)]. Children whose families were not subjected to political violence were more likely to have high levels of parental nurturing compared to those whose families were exposed to political violence [OR = 1.93, 95%CI (1.38–2.70)].

**Table 3:** Multivariate analysis for levels of parental nurturing

Predictor		***P*** value	Adjusted OR	95% CI	
				Lower	Upper
Gender (child)		0.67	1.07	0.77	1.50
Child’s age (years)	10–12	<0.001	1		
	Less than 6	<0.001	3.07	1.90	4.96
	6–9	<0.001	2.61	1.59	4.28
Child index within family	Only child/first child	<0.001	1		
	Middle child	0.37	0.75	0.39	1.42
	Last child	0.01	2.20	1.20	4.03
Child disability		0.01	1.95	1.17	3.25
Child discipline		<0.001	2.16	1.54	3.03
Locality	Camp	<0.001	1		
	Urban	0.01	2.25	1.18	4.29
	Rural	0.72	1.12	0.62	2.03
Region	South WB	<0.001	1		
	North WB	<0.001	4.59	3.04	6.92
	Center WB	0.26	1.28	0.83	1.96
Political violence		<0.001	1.93	1.38	2.70

## Discussion

We used secondary data representative of Palestinian children living in the WB of the oPt based on the Palestinian Census Projections of 2007 to estimate levels of parental nurturing among children aged 0 to 12 years and to uncover some of the associated social, economic and political factors. The Palestinian mothers living in the West Bank reported high levels of parental nurturing. Parental nurturing was found to be influenced by the social, economic, and political conditions the Palestinians are enduring. Child discipline was negatively associated with parental nurturing. These findings are in line with our proposed hypotheses. Overall, 19.9% of the children were at risk of receiving low levels of parental nurturing.

The result indicated that younger children below 6 years old were more likely to have high levels of parental nurturing compared to older children. This finding is supported by study of Chen and Liu which indicated a change in parenting practices at different stages of the child’s development [45]. McNally and colleagues attributed this to changes in children’s dependency, and emotional and academic support required from their families [46]. In 1984, Roberts and colleagues reported that when children grow up and move toward adolescence, they are more likely to have greater degree of autonomy and freedom [47]. As a result the parent’s role in control, involvement, supervision and interaction with their children is reduced, leading to changes in parental nurturing levels [20, 47].

Children who were last in the family were more likely to have high levels of parental nurturing. Indeed, research has shown that parental practices and responses differ by family size and the ordinal position of the child in the family [20]. Kidwell reported that when more births occur in the family, the first child’s central position and opportunities to have considerable attention, affection, and support is reduced leading to lower levels of parental nurturing [48]. This could also be a function of age. The last child is the youngest in the family and it could well be that attention and communication, with parents is highest among last children. Indeed, a local Palestinian saying about the last child describes the child as ‘the last in the bunch of grapes or clusters’, understood as the last child receiving most attention and being most loved.

Nondisabled children were more likely to have high levels of parental nurturing. A meta-analysis conducted to check whether relationship between child parents and parenting practices differ in families who have children with chronic illnesses and those who do not reported similar findings [49]. Parenting practices and parent child relationship play an important role in behavioral adaptation in children with chronic illness including, for example, adherence to prescribed diabetes medication [50]. Additionally, Roberts and Lawton emphasized that disabled children need extra parental care and efforts compared to healthy children, leaving insufficient time and energy for optimum parental nurturing [51]. Also, child disability adds economic burden on parents causing stress which interferes with parental affection and care [52]. High levels of stress among parents may lead to depression known to negatively affect parental nurturing [53, 54].

Children exposed to low to medium levels of disciplinary methods were more likely to have high levels of parental nurturing. In 2001, Wade and Kendler reported that physical discipline was inversely associated with parental nurturing a finding which endorses our finding [55]. In a review of the literature, Walters and Stinnet concluded that parents who showed acceptance, support and love to their children yielded better social and emotional child development, while rejection and punishment negatively affected the child’s emotional and social development [20]. Parents who reported using more abusive methods of punishment were less likely to have positive interaction with their children and more likely to utilize disciplinary approaches [56].

Parental nurturing was found to vary by type of locality. Children from urban places were more likely to have high levels of parental nurturing compared to children from Palestinian refugee camps. Palestinian refugee camp dwellers are disadvantaged and often suffer from poverty, unemployment, unhealthy and overcrowded housing conditions [57, 58]. High levels of psychological distress reported among refugees have been attributed to the deteriorated social, economic and political environment [59–62]. The way camp residents live where there is a lack of basic human living requirements such as personal and family safety, economic prosperity, good housing, and food security [63, 64] are believed to be the main causes of the psychological distress and depression that interferes with levels of parental nurturing [54].

Parental nurturing was associated with region. Children living in North WB were four times as likely to have high levels of parental nurturing compared to those living in South WB. This was a major finding in our study. Underlying causes of this pattern of nurturing are not well known and further investigations are required to identify main causes of this finding. However, economic, political, and developmental conditions could be an explanation. The southern region of the West Bank is the most populated region of the west bank, and the Hebron district in particular is known to have the lowest levels of educational attainment among its population including the highest levels of illiteracy, the highest mean number of children, the lowest level of access to safe drinking water, and the lowest proportion of computer access at home [65, 66]. These results generally correspond to a 2002 study which demonstrated that the southern region in general and the Hebron district in particular is less exposed to the outside world, with more conservative social and cultural characteristics than the rest of the West Bank which can help explain the results of this study [67]. Furthermore, other investigations have reported high levels of exposure to violence in the Hebron district [68]. Overall, the use of discipline, large family sizes and crowded homes were more prevalent in south WB, while the educational levels of North West bankers were higher than those from the south, which can also help explain the results.

Children whose families were not subjected to political violence were more likely to have high levels of parental nurturing. Parental nurturing cannot be understood without proper investigation of the social, economic and political context [69]. Those who experience political violence are at greater risk of developing fear and distress [70]. In the oPt political violence was found to be associated with increasing occurrence of mental health problems including trauma and stress [71]. In addition, exposure to political violence has a deleterious effects on family function leading to economic burden, stress, somatization, anxiety [72] and increases the risk of child abuse [73]. Such factors could explain low levels of parental nurturing among families experiencing political violence.

### Study limitations

This is a cross sectional study which demonstrates association but not causation. Palestinian children living in the Gaza Strip which is characterized by different social, economic, and political context were not included in this study. Other important variables like maternal health were also not included in the study. This is important to investigate since maternal health problems such as physical illness, stress and other mental health problems compromises their capacity to nurture their children adequately [7, 74]. Some of the discipline questions may have been under-reported by mothers because of social sensitivity and worry that they would be blamed by family and community. There are several limitations to the use of the ICAST-P tool, which includes the above point about maternal health, relations with husband, maternal exposure to violence at home, community and beyond. However, there is a limit to the number of questions which one can include in a study. In addition, the advantage of using the ICAST-P is that we are able to compare internationally.

## Conclusions

Although Palestinian mothers showed high levels of parental nurturing as we expected, around one-fifth of the children are still at risk of receiving low levels of parental nurturing. Risk factors identified in this study include age of the children, their ordinal position in the family, disabled children, refugee camp dwellers, South WB children, and among families exposed to political violence. Some of these associated factors are modifiable with intervention programs focused on the identified priority groups of children. This could include improving the daily living conditions of families, providing social, and economic support to disadvantaged groups such as those living in the refugee camps, which has been shown to improve the overall levels of parental nurturing [7]. Our results nevertheless emphasize the impact of social, economic and political dimensions on health. More attention should be paid to improve daily living conditions and health needs of children at risk. Ultimately, a just political solution to the Palestine question especially as this relates to Palestinian refugees is also of essence.

## Data Availability

Data and materials are available from the authors upon a reasonable request.

## Appendix 1: Parental nurturing questions

1. Did you/husband/care taker tell this child that you loved him during the past 12 months?
2. Did you ever show love to this child during the past 12 months?
3. Do you think this child knows that he/she is loved?

## Appendix 2: Physical and Psychological abuse questions (ICAST-P)

V10. “Shook him/her”.

V11. “Hit him or her on the buttocks with an object such as a shoe, stick, broom, cane or belt”.

V12. “Hit elsewhere (not buttocks) with an object such as a shoe, stick, broom, cane or belt”.

V14. “Twisted his/her ear”.

V15. “Hit him/her on head with knuckle or back of the hand”.

V16. “Pulled his/her hair”.

V17. “Threatened to leave or abandon him/her”.

V18. “Shouted, yelled, or screamed at him/her”.

V19. “Threatened to invoke ghosts, ghoul or evil spirits, or harmful people”.

V20. “Kicked him/her with a foot”.

V21. “Put chili pepper, hot pepper, or spicy food in mouth (to cause pain)”.

V22. “Forced him/her to kneel or stand in a manner that result in pain”.

V23. “Cursed him/her (Son of a …)”.

V24. “Spanked him/her on the bottom with bare hand”.

V25. “Choked him/her or squeezed his or her neck with hands (or something else)”.

V26. “Threatened to kick out of house or send away for a long time”.

V27. “Locked out of the house”.

V29. “Insulted him/her by calling [name] dumb, lazy, or other names like that”.

V30. “Pinched him/her”.

V31. “Slapped on face or back of head”.

V32. “Refused to speak to him/her”.

V33. “Withheld a food meal as punishment”.

V34. “Used a hand or pillow to cover the mouth and nose to prevent breathing (smother)”.

V35. “Burned, scalded or branded him/her”.

V36. “Hit him or her over and over again with object or fist (“beat-up”)”.

V37. “Threatened him/her with a knife or gun”.

V38. “Locked him or her in a dark room”.

V39. “Used public humiliation to discipline him or her”.

## Abbreviations

WB: West Bank
oPt: Occupied Palestinian territory
PCBS: The Palestinian Central Bureau of Statistics
ISPCAN: The International Society for Prevention of Child Abuse and Neglect
ICASTs: ISPCAN Child Abuse Screening Tools
ICAST-P: ISPCAN Child Abuse Screening Tool (parents version)
ICAST-C: ISPCAN Child Abuse Screening Tool (children version)
ICAST-R: ISPCAN Child Abuse Screening Tool (retrospective reporting)
OR: Odds ratio
CI: Confidence interval
SD: Standard deviation
